# Visual food stimulus changes resting oscillatory brain activities related to appetitive motive

**DOI:** 10.1186/s12993-016-0110-3

**Published:** 2016-09-26

**Authors:** Takahiro Yoshikawa, Masaaki Tanaka, Akira Ishii, Yoko Yamano, Yasuyoshi Watanabe

**Affiliations:** 1 Department of Sports Medicine, Osaka City University Graduate School of Medicine, 1-4-3 Asahi-machi, Abeno-ku, Osaka, Osaka 545-8585 Japan; 2 Department of Physiology, Osaka City University Graduate School of Medicine, 1-4-3 Asahi-machi, Abeno-ku, Osaka, Osaka 545-8585 Japan; 3 RIKEN Center for Life Science Technologies, 6-7-3 Minatojima-minamimachi, Chuo-ku, Hyogo 650-0047 Japan

**Keywords:** Resting brain activity, Magnetoencephalography (MEG), Insula, Dorsolateral prefrontal cortex (DLPFC), Orbitofrontal cortex (OFC), Frontal pole

## Abstract

**Background:**

Changes of resting brain activities after visual food stimulation might affect the feeling of pleasure in eating food in daily life and spontaneous appetitive motives. We used magnetoencephalography (MEG) to identify brain areas related to the activity changes.

**Methods:**

Fifteen healthy, right-handed males [age, 25.4 ± 5.5 years; body mass index, 22.5 ± 2.7 kg/m^2^ (mean ± SD)] were enrolled. They were asked to watch food or mosaic pictures for 5 min and to close their eyes for 3 min before and after the picture presentation without thinking of anything. Resting brain activities were recorded during two eye-closed sessions. The feeling of pleasure in eating food in daily life and appetitive motives in the study setting were assessed by visual analogue scale (VAS) scores.

**Results:**

The γ-band power of resting oscillatory brain activities was decreased after the food picture presentation in the right insula [Brodmann’s area (BA) 13], the left orbitofrontal cortex (OFC) (BA11), and the left frontal pole (BA10). Significant reductions of the α-band power were observed in the dorsolateral prefrontal cortex (DLPFC) (BA46). Particularly, the feeling of pleasure in eating food was positively correlated with the power decrease in the insula and negatively with that in the DLPFC. The changes in appetitive motives were associated with the power decrease in the frontal pole.

**Conclusions:**

These findings suggest automatic brain mechanics whereby changes of the resting brain activity might be associated with positive feeling in dietary life and have an impact on the irresistible appetitive motives through emotional and cognitive brain functions.

## Background

Today’s lifestyle provides ample opportunities for pleasurable but excessive food intake [1], which often leads to obesity and becomes a considerable health threat in susceptible individuals by raising the risk of chronic diseases such as diabetes mellitus, hypertension, heart disease, fatty liver, sleep apnea, and certain forms of cancer [2, 3]. Another health issue associated with modern dietary lifestyles is related to the physiological and psychological reductions in food intake that can be important contributors to sarcopenia in older individuals [4] as well as malnutrition in adolescents and young adult women [5]. Accordingly, from a public health perspective, it is imperative to clarify the control mechanisms involved in eating behaviors and to develop new strategies to encourage the consumption of proper nutrition. In particular, it is crucial to understand the neurobiological mechanisms by which the decision to start or stop eating comes about [6].

Eating behavior is affected by various physiological determinants including homeostatic requirements such as nutritional deficits, and is regulated by metabolic and neuroendocrine networks integrating central nervous pathways with signals from the periphery [7]. However, it is also known that human eating behaviors largely depend on cognitive (attention, learning, memory, and decision-making), sensory (visual, olfactory, taste, and somatosensory), and behavioral (motivational) processes [8, 9]. Additionally, fluctuations in mood and emotion can affect food choices and quantities [10]. Human eating behavior is thus extraordinarily complex, and disturbances in eating behaviors are difficult to treat.

Neuroimaging techniques have classically been employed to elucidate the neurological bases of eating behaviors. Positron emission tomography (PET) and functional magnetic resonance imaging (fMRI) have been utilized most frequently in research concerning eating behaviors. Hemodynamic changes are assessed as indicators of the changes of neural activation relating to cognition, emotion, and behaviors in various experimental conditions, including the fed state and stimuli (visual, olfactory, gustatory, and food intakes) [11]. In contrast, magnetoencephalography (MEG) monitors electrophysiological activities inside the brain by measuring electromagnetic fields using electric or magnetic sensors over the scalp surface [12–14]; it allows a quantitative assessment of oscillatory components in measured data, and synchronization of oscillatory neuronal firing represents a physiological coding mechanism to bind together spatially separated populations of neurons [15, 16]. Thus, brain activities are characterized by the presence of more and less regular oscillations in various frequency bands [16]. For example, a decrease of the α-band power suggests deactivated interaction between local negative feedback circuits in the thalamus and the cortex, while a decrease of the γ-band power indicates deactivated information processing reflecting reduced rapid synchronization among local brain areas [17, 18].

Recently, we applied the MEG analyses to research pertaining to the time course of neural processes for appetitive motives and self-control immediately after visual exposure to food pictures [19–21]. While most previous studies investigated ongoing neural networks during a sensory presentation relating to food, it is also valuable to focus on differences in resting brain activities before and after a series of visual stimuli using food images, by assessing changes in synchronization of oscillatory neuronal firing between these two conditions. Such differences, which could not be assessed by using fMRI or PET, might characterize involuntary residual effects in the resting brain activities that may persist after a cessation of visual food stimulation, and the lasting effects might transiently modify dietary emotion and cognition such as the appetitive motives or decisions to eat, and further impact the feelings of pleasure in a real dietary life in individuals who cannot help consuming too much or in those who cannot consume a sufficient amount of food.

In the present study, we measured resting brain activities by using MEG in fasting individuals who closed their eyes for 3 min before and after watching various food pictures presented every 2 s for 5 min. Throughout the experiment, the contents of the pictures were not disclosed to the study participants in advance, and they were instructed not to think of anything, including the pictures on the screen. We tried to identify the brain areas related to the residual effect on the resting brain activities by examining the differences in the oscillatory power between 2 eye-closed conditions, before and after the presentation of food pictures, using the time–frequency analyses of MEG. Next, we tried to determine whether the oscillatory power changes are associated with a transient change in appetitive motives spontaneously elicited by the visual presentation of food items. Additionally, in order to confirm the validity of our results in a real-life situation, we assessed the impact of the oscillatory power differences on practical factors related to appetitive motives and eating habits such as the feeling of pleasure in eating food in daily life. We hypothesized that, based on the previous literature when viewing food [11, 19–21], a short duration of visual food stimuli has a considerable impact on resting oscillatory brain activities which manifest as changes of powers across various time–frequency bands in emotional and cognitive brain areas.

## Methods

### Participants

In total, fifteen healthy male volunteers with normal body habitus [age, 25.4 ± 5.5 years; height, 171.3 ± 6.9 cm; body weight, 66.3 ± 11.7 kg; body mass index (BMI), 22.5 ± 2.7 kg/m^2^ (mean ± SD)] were enrolled. Participants with a history of mental or neurological disorders, and those taking chronic medications that affect the central nervous system were excluded. All of the participants had normal or corrected-to-normal visual acuity and were right-handed. The study protocol was approved by the Ethics Committee of Osaka City University (license number 2811), and all participants provided written informed consent to participate in the study and were monetarily compensated. All procedures were done according to the research ethics of the Helsinki Declaration of 1975, and the applicable revisions at the time of the investigation [22].

### Experimental design

**Fig. 1:**
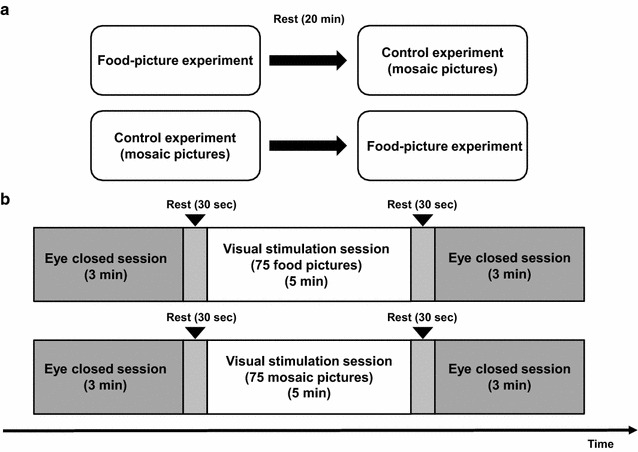
Experimental design (**a**) and the procedure of experimental sessions (**b**). Participants were enrolled in a randomized study consisting of 2 crossover experiments (food-picture and control experiments), and asked to watch a series of pictures for 5 min (visual stimulation session) and to close their eyes for 3 min before and after the picture presentation (eye-closed sessions). Pictures of food items were presented as visual stimuli during the food-picture experiment, and mosaic pictures of food items were presented as visual stimuli during the control experiment. The contents of pictures were not disclosed to the study participants in advance

The participants were enrolled in a randomized study consisting of two crossover experiments (food-picture and control experiments) (Fig. 1a). For 1 day before the visit, they were instructed to finish dinner by 9:00 p.m. and to fast overnight (they were only allowed to drink water), to avoid intensive physical and mental activities, and to maintain normal sleeping hours. After they arrived at the laboratory in the morning, they were asked to report their physical condition including hunger. They rated their subjective level of hunger on a 5-point Likert-type scale ranging from 1 (*Yes, I am very hungry*) to 5 (*No, I am not hungry at all*). In addition, they were asked to rate their feeling of pleasure in eating food in daily life, using a unidimensional visual analogue scale (VAS), ranging from none (0 mm) to maximum (100 mm). Then, they were instructed to watch a series of pictures for 5 min (visual stimulation session) and to close their eyes for 3 min before and after the picture presentation (eye-closed sessions) (Fig. 1b). The contents of the pictures were not disclosed to the study participants in advance. The intersession intervals were set at approximately 30 s. Brain activities were recorded using MEG during these two eye-closed sessions. The pictures of food items were presented as visual stimuli during the food-picture experiment. In addition, the mosaic pictures created from the same pictures of food items were used as visual stimuli during the control experiment. During the visual stimulation sessions, while in the supine position on a bed, the participants were requested to keep both eyes open and to fixate on a central point and to view the screen. During eye-closed sessions, they were instructed to close their eyes while in the same position as the visual stimulation session. Throughout the experiment, they were instructed not to think of anything, including the pictures on the screen. They were asked to rate their appetitive motives, using a VAS, ranging from none (0 mm) to maximum (100 mm) for each of the eye-closed and visual stimulation sessions in both experiments. This study was conducted in a quiet, temperature-controlled magnetically shielded room at Osaka City University Hospital.

### Stimulus presentation

**Fig. 2:**
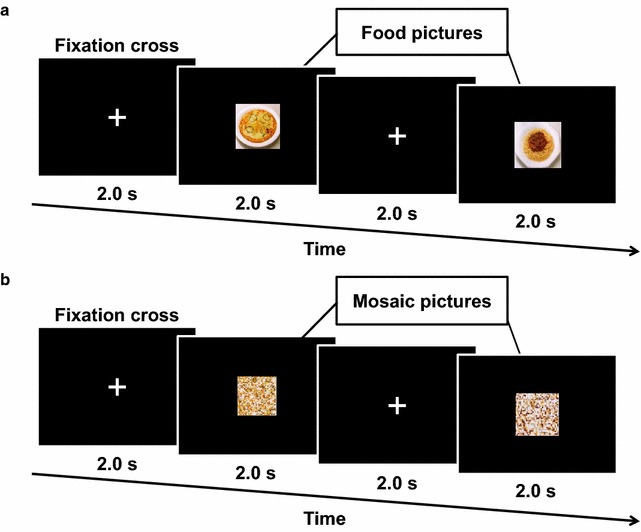
The time course of stimulus presentations. A series of 75 color food pictures, consisting of 15 food items (**a**), and a series of 75 mosaic pictures, consisting of 15 mosaic pictures of food (**b**), were used. The order of the picture presentation was randomized for each series, and the sequences of pictures for presentation were randomly assigned for each participant. The mosaic pictures created from the same pictures of food items were used as visual stimuli in the same sequences as the food pictures during the control sessions. The original images used to generate the mosaic pictures were not disclosed to the study participants. Each picture was presented for 2.0 s followed by a fixation cross for 2.0 s

Visual stimulus presentation was performed similarly as described in our previous study [20]. Briefly, each visual stimulation session consisted of 75-picture sets of a 2-sec stimulation period followed by a 2-sec inter-stimulus interval (Fig. 2a, b). Fifteen pictures of typical modern Japanese food items were used, including steak, hamburger, fritter, chicken nugget, French fry, pizza, spaghetti, ice cream, and noodles [23]. After the experiment, each participant was asked to rate each picture for food preference in order to confirm that disliked food items were not included. Each picture was used five times to construct the 75-picture set. The mosaic images of the original photographs (15 food items) were also used to control for luminance, color, and local features [24, 25]. Mosaic pictures were made using commercial software (Adobe^®^ Photoshop Elements 6.0, Adobe Systems Inc., San Jose, CA). All of the food pictures were divided into a 30 × 30 grid and randomly reordered by a constant algorithm. This rearrangement made each picture unrecognizable as food. The original images used to generate the mosaic pictures were not disclosed to the study participants. The sequences of pictures for presentation were randomly assigned for each participant, but the same sequences were used for both food-picture and control experiments. These pictures were projected on a screen placed in front of the participants’ eyes using a video projector (PG-B10S; SHARP, Osaka, Japan). The viewing angle of each picture was 18.4 × 14.0°.

### MEG data acquisition

MEG data acquisition was performed using a whole-head type MEG system (MEG vision; Yokogawa Electric Corporation, Tokyo, Japan) with 160 channels. The signals were continuously recorded at a sampling rate of 1000 Hz in gradiometers 15.5 mm in diameter and 50 mm in baseline with a 0.3 Hz high-pass filter.

Structural MR images were obtained using a Philips Achieva 3.0TX (Royal Philips Electronics, Eindhoven, The Netherlands). Before MRI scanning, 5 adhesive markers (Medtronic Surgical Navigation Technologies Inc., Broomfield, CO) were attached to the skin of each participant’s head (the first and second ones were located 10 mm in front of the left tragus and right tragus, the third at 35 mm above the nasion, and the fourth and fifth at 40 mm right and left of the third one), which were the former positions of MEG localization coils. MEG data were coregistered on MRI scans using information obtained from these markers and MEG localization coils.

### MEG data analyses

MEG data analyses were performed similarly as described in our previous study [20]. Briefly, MEG signal data were analyzed offline after analogue-to-digital conversion. Magnetic noise originating outside the shield room was eliminated by subtracting the data obtained from reference coils using MEG 160 (Yokogawa Electric Corporation, Tokyo, Japan). Artifact rejection of MEG data was performed through careful visual artifact detection before band pass filtering and averaging. The MEG data were band-pass filtered by a fast Fourier transform using frequency trend (Yokogawa Electric Corporation) to obtain time–frequency band signals using a software, brain rhythmic analysis for MEG (BRAM; Yokogawa Electric Corporation) [26]. After the band pass filtering, the MEG data were split into segments of 1000 ms in length, and the segments were averaged.

Localization and intensity of the time–frequency power changes were assessed by BRAM software, which used narrow-band adaptive spatial filtering methods as an algorithm [26]. We used statistical parametric mapping (SPM8, Welcome Department of Cognitive Neurology, London, UK) in Matlab (Mathworks, Sherbon, MA) to analyze the MEG data. We initially performed normalization to the Montreal Neurological Institute (MNI) template of T1-weighed images [27] and smoothing (the normalized MEG data were filtered with a Gaussian kernel of 20 mm [full-width at half-maximum] in the x, y, and z axes [voxel dimension was 5.0 × 5.0 × 5.0 mm]). The whole brain oscillatory powers for δ-band (1–4 Hz), θ-band (4–8 Hz), α-band (8–13 Hz), β-band (13–25 Hz), and γ-band (25–50 Hz) during the eye-closed session were measured on a region-of-interest basis. To investigate the alterations of the brain activities during the resting eye-closed condition after the visual stimulation session, we analyzed two contrast images: image after the visual stimulation of food pictures > image before the visual stimulation of food pictures; and image after the visual stimulation of mosaic pictures > image before the visual stimulation of mosaic pictures. The two contrast images were submitted to one sample *t* test on a voxel-by-voxel basis [28]. The threshold of individual analyses was set at *P* < 0.05 (corrected for multiple comparisons). Individual data were incorporated into a random-effect model so that inferences could be made at a population level [28]. The threshold of group analyses was set at *P* < 0.05 (corrected for multiple comparisons). Anatomical localizations of significant voxels within clusters were expressed in the form of MNI stereotactic spatial coordinates (x, y, z). In addition, these coordinates were converted to corresponding Brodmann’s area (BA) by using the Talairach Daemon software [29].

### Statistical analyses

Data are expressed as mean ± SD. Two-way analyses of variance followed by paired t test with Bonferroni correction were performed to examine the differences in VAS scores for appetitive motives among different sessions and experiments. Pearson’s correlation analyses were used to examine whether the changes in the power of resting oscillatory brain activities by visual food presentation might be associated with subjective variables. All *P* values were two-tailed, and values less than 0.05 were considered statistically significant. Statistical analyses were performed using the SPSS 18.0 software package (SPSS, Inc., Chicago, IL).

## Results

### Decrease of resting oscillatory brain activities after visual food stimulation

Table 1 summarizes the results of spatial filtering analyses that show brain regions with a greater decrease of resting oscillatory band power during the eye-closed session after a cessation of visual food stimulation relative to that before the stimulation. These include the following 4 regions: (1) the right insula (BA13); (2) the right middle frontal gyrus (BA9 and 46) corresponding to the dorsolateral prefrontal cortex (DLPFC); (3) the left subcallosal gyrus (BA11) corresponding to the orbitofrontal cortex (OFC); and (4) the left superior frontal gyrus (BA10) corresponding to the frontal pole.

**Table 1:** Brain regions that show a greater decrease of resting oscillatory band power during the eye-closed session (after a cessation of visual food stimulation) relative to that before the stimulation

Location	Frequency band (Hz)	Side	BA	MNI coordinate (mm)			Z value
				x	y	z	
DLPFC	4–8	R	9	52	28	40	3.78
OFC	8–13	L	11	−38	38	−15	3.94
DLPFC	8–13	R	46	42	48	20	3.57
Insula	25-50	R	13	42	−7	5	3.36
Insula	25–50	R	13	42	8	0	3.35
Frontal pole	25–50	L	10	−23	53	−5	2.93
OFC	25–50	L	11	−13	28	−10	2.76
DLPFC	25–50	R	9	27	53	40	2.71

x, y, z: stereotaxic coordinates of peak of activated clusters

*BA* Brodmann’s area, *MNI* Montreal Neurological Institute, *DLPFC* dorsolateral prefrontal cortex, *OFC* orbitofrontal cortex, *L* left, *R* right

Random-effect analyses of 15 participants (*P* < 0.05, corrected for multiple comparisons at the voxel level)

### Rating scores of hunger before recordings of MEG, those of feeling of pleasure in eating food in daily life, and those of appetitive motives elicited spontaneously by presentation of food pictures—association with the decrease of resting oscillatory brain activities

**Fig. 3:**
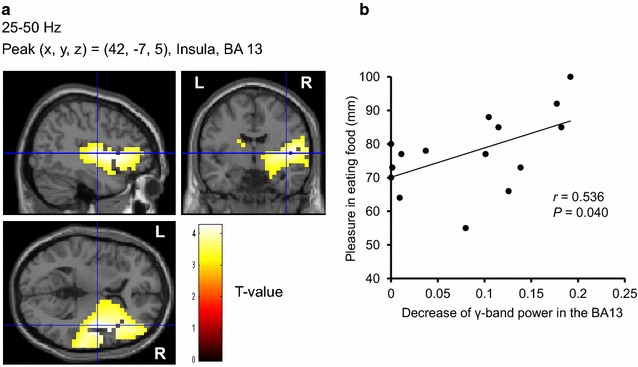
Localization and intensity of the decrease of γ-band power during the eye-closed session after the cessation of the visual stimulation session, relative to the band power before the start of the visual stimulation session in the insula (BA13) (**a**) (random effect analyses of 15 participants, *P* < 0.05, corrected for multiple comparisons at voxel level) and association of the power decrease with the feeling of pleasure in eating food in daily life (**b**). **a** The results are superimposed on high-resolution MRIs averaged across all the participants. Sagittal (*upper left*), coronal (*upper right*), and axial (*lower left*) sections are shown. The *color bar* indicates T values. **b** Linear regression line, Pearson’s correlation coefficient (*r*), and *P* value are shown. *BA* Brodmann’s area, *R* right, *L* left

**Fig. 4:**
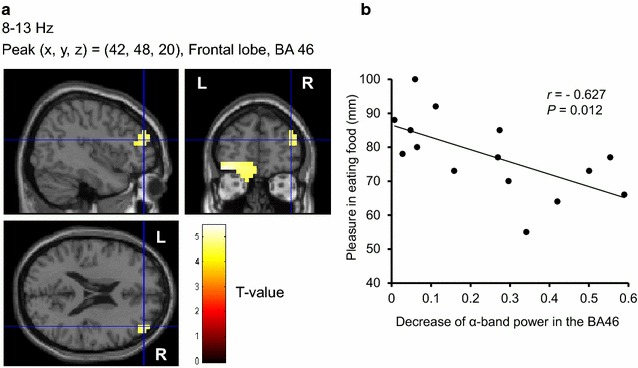
Localization and intensity of the decrease of α-band power during the eye-closed session after the cessation of the visual stimulation session, relative to the band power before the start of the visual stimulation session in the DLPFC (BA46) (**a**) (random effect analyses of 15 participants, *P* < 0.05, corrected for multiple comparisons at voxel level) and association of the power decrease with the feeling of pleasure in eating food in daily life (**b**). **a** The results are superimposed on high-resolution MRIs averaged across all the participants. Sagittal (*upper left*), coronal (*upper right*), and axial (*lower left*) sections are shown. The *color bar* indicates T values. **b** Linear regression line, Pearson’s correlation coefficient (*r*), and *P* value are shown. *BA* Brodmann’s area, *DLPFC* dorsolateral prefrontal cortex, *R* right, *L* left

**Fig. 5:**
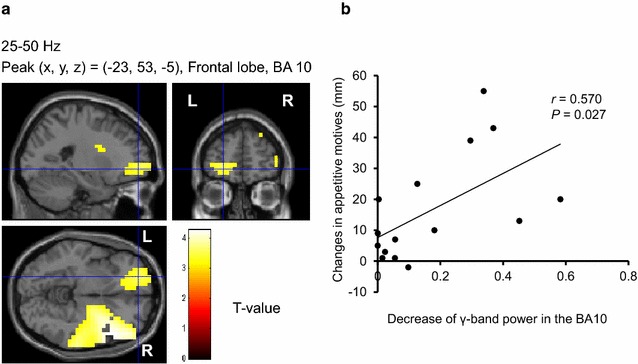
Localization and intensity of the decrease of γ-band power during the eye-closed session after the cessation of the visual stimulation session, relative to the band power before the start of the visual stimulation session in the frontal pole (BA10) (**a**) (random effect analyses of 15 participants, *P* < 0.05, corrected for multiple comparisons at voxel level) and association of the power decrease with the changes in appetitive motives during and after visual food stimulation assessed by 100 mm VAS scores (**b**). **a** The results are superimposed on high-resolution MRIs averaged across all the participants. Sagittal (*upper left*), coronal (*upper right*), and axial (*lower left*) sections are shown. The *color bar* indicates T values. **b** Linear regression line, Pearson’s correlation coefficient (*r*), and *P* value are shown. *BA* Brodmann’s area, *R* right, *L* left, *VAS* visual analogue scale

**Fig. 6:**
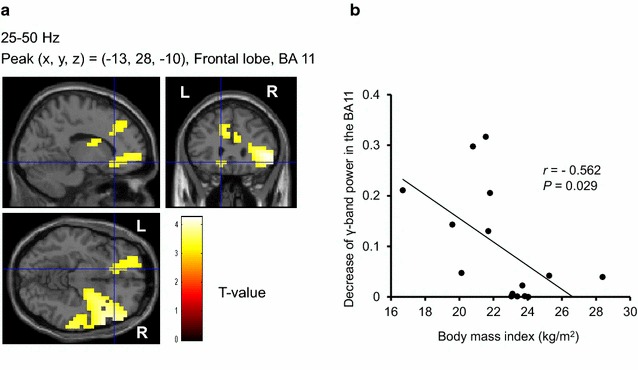
Localization and intensity of the decrease of γ-band power during the eye-closed session after the cessation of the visual stimulation session, relative to the band power before the start of the visual stimulation session in the OFC (BA11) (**a**) (random effect analyses of 15 participants, *P* < 0.05, corrected for multiple comparisons at voxel level) and association of the power decrease with body mass index (**b**). **a** The results are superimposed on high-resolution MRIs averaged across all the participants. Sagittal (*upper left*), coronal (*upper right*), and axial (*lower left*) sections are shown. The *color bar* indicates T values. **b** Linear regression line, Pearson’s correlation coefficient (*r*), and *P* value are shown. *BA* Brodmann’s area, *OFC* orbitofrontal cortex, *R* right, *L* left

Before the MEG recordings, all of the participants rated their subjective level of hunger as almost excessive [1.7 ± 0.6 (mean ± SD) on a 5-point Likert-type scale]. The mean VAS score of pleasure in eating food in daily life assessed was 78 ± 12 mm. They had considerable motives to eat during the visual stimulation in the food-picture experiment (Table 2). The subjective VAS scores for appetitive motives in the food-picture experiment were significantly higher than those in the control experiment (*P* < 0.001, main effect of experiment assessed by two-way analysis of variance), and the scores were significantly increased by the visual stimulation of food items (*P* < 0.001, main effect of session assessed by two-way analysis of variance; *P* < 0.001, assessed by post hoc paired *t*-test with Bonferroni correction). Correlation analyses revealed that the VAS scores for pleasure in eating food in daily life were positively associated with the decrease of the γ-band power of resting oscillatory brain activities in the right insula (BA13) (*r* = 0.536, *P* = 0.040) (Fig. 3), and were inversely associated with that of α-band of resting oscillatory brain activities in the right DLPFC (BA46) (*r* = −0.627, *P* = 0.012) (Fig. 4), respectively. Significant correlations were found between the difference in the scores for appetitive motives during and after the food picture stimulation and the decrease of the γ-band power of resting oscillatory brain activities in the left frontal pole (BA10) (*r* = 0.570, *P* = 0.027) (Fig. 5). In addition, the decrease of the γ-band power of resting oscillatory brain activities in the left OFC (BA11) was negatively correlated with BMI (*r* = −0.562, *P* = 0.029) (Fig. 6).

**Table 2:** Subjective levels of appetitive motive

	Eye-closed session (before stimulation)	Visual stimulation session	Eye-closed session (after stimulation)
Food-picture experiment	34 ± 25	79 ± 17*	62 ± 26^†,‡^
Control experiment	37 ± 25	33 ± 28	32 ± 23

Data are expressed as mean ± SD (n = 15)

Participants were asked to rate their appetitive motive by using a visual analogue scale, ranging from none (0 mm) to maximum (100 mm)

Two-way analyses of variance followed by paired *t* test with Bonferroni correction were performed. Main effect of experiment, *P* < 0.001; main effect of session, *P* < 0.001; experiment × session interaction effect, *P* < 0.001

* *P* < 0.001 [visual stimulation session vs. eye-closed session (before stimulation)]

^†^ *P* = 0.007 [visual stimulation session vs. eye-closed session (after stimulation)]

^‡^ *P* = 0.001 [eye-closed session (before stimulation) vs. eye-closed session (after stimulation)]

## Discussion

The present study demonstrates that a short duration of visual food stimuli has a considerable impact on resting oscillatory brain activities during eye closure in various emotional and cognitive brain areas such as the right insula (BA13), the right DLPFC (BA46), the left frontal pole (BA10) and the left OFC (BA11). Interestingly, the feelings of pleasure in eating food in daily life were positively associated with oscillatory power decreases in the insula, and negatively with those in DLPFC. Furthermore, in spite of the instruction not to think of anything including the pictures on the screen, the participants had appetitive motives spontaneously elicited by food picture stimulation, and the changes in appetitive motives were significantly related to the power decrease of the resting oscillatory brain activities in the left frontal pole.

One of the major findings is a significant decrease of the γ-band power of resting oscillatory brain activities in the right insula (BA13). Previous studies have suggested that this brain region performs a diversity of functions, some of which are linked to dietary lifestyle. For example, in our previous studies, equivalent current dipoles (ECDs) assessed by MEG were detected in the insula immediately (approximately 300 ms) after the onset of food picture presentation in response to viewing these pictures under the instruction to have appetitive motives in the fasting [19] or postprandial conditions [21]. These findings suggest the possible involvement of insula in the neural processes of the motivations to eat. Although the present study did not focus on the time course of the immediate neural responses after visual food stimuli nor on the neural responses with having motivation to eat volitionally, a new evidence has surfaced that would support the involvement of insular activity even during the absence of visual food stimuli. Furthermore, the present study demonstrates the positive association of the resting brain activity in the insula with the feelings of pleasure in eating food in daily life. In line with this, functions of insula include the representation of pleasantness of flavor [30] and the control of habituation in food-related stimulation [31]. Considering these previous evidences, it is possible that the present residual insular activity as observed even after a cessation of stimulation might play a role in the formation of the positive emotional aspect (feeling of pleasure) in eating food and subsequently modify the habituation process of eating behaviors in daily dietary life.

Another finding in the present study was a significant decrease of the α-band power of resting oscillatory brain activities in the right DLPFC (BA46). In addition, the present study showed the inverse association of the resting brain activities in this brain region with the feelings of pleasure in eating food in daily life. In other words, the changes in the resting brain activity in the DLPFC tends to be more pronounced in individuals with less pleasure associated with eating food. Most of the previous studies demonstrated that the DLPFC plays a major role in the self-control in eating food as indicated by an association of the brain activities with the measure of ordinary eating behaviors such as cognitive restraints assessed using questionnaires [32, 33]. The present finding might imply that cognitive self-control of the desire for food might affect the expression of positive emotion to eat such as pleasure in eating food via this brain mechanism. Combined with the results in insular activity, the transient visual food stimuli could disturb the emotional and cognitive domains of the resting brain activities even in the absence of concurrent visual stimuli.

In the present study, it is important to note that the participants were asked not to think of anything about pictures in the stimulus presentation. Nevertheless, their appetitive motives were spontaneously elicited as assessed using the VAS, and interestingly, the changes in appetitive motives were positively associated with the power decrease of the resting oscillatory brain activities in the left frontal pole. The frontal poles have been reported to play roles in memory retrieval [34–36] as well as in memory encoding and recognition [36–38]. In addition to the functions of retrospective memory, the frontal poles are more involved with thinking about the future than about the past [39]. Based on the observed association with temporal fluctuations in appetitive scores of VAS, the frontal pole plays a role in subsequent planning, such as thinking about what to eat after a cessation of visual exposure of food items.

Previous neuroimaging studies have been inconsistent regarding structural and functional alterations in obesity. In particular, the OFC is one of major components of reward circuitry related to overeating palatable food and development of obesity [11, 40, 41]. In contrast, the OFC is reported to be an important structure in the termination of food intake, and disturbance in this function could contribute to overconsumption of food in obesity [42]. The present study demonstrated that the resting brain activity observed in the OFC is negatively correlated with the BMI. The range of the BMI in the present participants was less than 30 kg/m^2^. Its physiological and clinical implications warrant further studies, particularly in more obese individuals.

Accumulating evidence suggests that visual afferent signals provide information to the central nervous system for appetite regulation even before food is ingested, and the limbic, paralimbic, and frontal brain circuits play important roles in neural processing during the visual stimuli in obese, healthy weight, and weight-loss populations [11, 43]. These brain regions are known to participate in emotional, salience, memory, reward, cognitive, and visual processing. In contrast, only a limited number of neuroimaging studies have addressed the residual effect of neural responses after the cessation of visual food stimulation like aftertaste [44]. Such a residual effect might be one of the important determinants of pleasure after a meal, possibly leading to a dietary learning process, irrespective of the type of sensation, and affect the decision to start or to stop eating subsequently [45]. Compared with traditional research, the present observation is unique because the essence of the study design was to examine the residual effect of neural responses to visual food stimuli by assessing the differences of the resting oscillatory brain activities before and after a series of visual presentations of food items. Collectively, the observed characteristics of the changes of resting brain activity before and after visual food stimulation might contribute to the formation of the feeling of pleasure in eating food and irresistible appetitive motives, and it might affect subsequent eating behaviors, possibly through emotional and cognitive functions including memory retrieval and future planning.

The present study has some potential limitations. First, we examined the brain activity in normal-weight young adults without apparent eating disorders during a fasted state. In order to clarify the impact of the changes of resting brain activities in general, further studies using similar MEG analytic methods in individuals with distorted eating habits and eating disorders in either the fasting state and/or during satiety will be needed. Second, the design of the present study is to assess brain activity caused by visual food cues. Since eating behavior can be evoked through multiple sensory systems, in order to generalize the results of our data, future studies using other sensory modalities are essential. Third, a small number of subjects were recruited in the present study. A large population study will be necessary to confirm the present results. Fourth, the present instruction not to think of anything, including the pictures on the screen, might force the study participants to attempt to suppress their thoughts and feelings more than required. The observed brain areas related to self-control systems like DLPFC might not be in accordance with the original study purpose to identify brain areas of the resting brain activities simply when one closes his eyes without thinking anything. Fifth, it is more likely that, after the visual stimulation session, the hungry participants were ‘replaying’ in their heads the pictures of food that they saw a couple of minutes earlier. It is well known that experiencing an actual sensory stimulus or imagining the sensory stimulus activates the same brain areas. Sixth, in the present study, we did not measure brain activity during the 5-min visual food stimulation. Since the brain areas activated and the power changes obtained during stimulation might correlate with the power changes and activation locations after stimulation, it will be needed to compare the brain activities among before, during and after the visual food stimulation. Seventh, the analyses included a one-sample test on two contrasts involving time following food and mosaic pictures. A 2 (time) × 2 (stimuli) interaction effect should be tested to determine the specificity of the effect. Lastly, we cannot draw conclusions about cause-and-effect relationships because of the cross-sectional nature of our data.

## Conclusions

The present findings raised the intriguing possibility that a series of visual food stimuli have a significant impact on resting oscillatory brain activities in the insula, DLPFC, OFC, and frontal pole. Since the changes of the resting brain activity are positively associated with positive emotion such as pleasure in eating food in daily life and appetitive motives, these changes are likely to determine subsequent eating behaviors. Although firm intention and conscious efforts are necessary for improvements in abnormal eating behavior and habits in a person’s dietary lifestyle, it is also important to devote considerable attention to the characteristics of the automatic or unconscious brain mechanics after food-related stimulation in order to develop efficient strategies for optimizing dietary lifestyle in people who fall into overeating and anorexia against their will.

## Abbreviations

BA: Brodmann’s area
BMI: body mass index
BRAM: brain rhythmic analysis for MEG
DLPFC: dorsolateral prefrontal cortex
fMRI: functional magnetic resonance imaging
MEG: magnetoencephalography
MNI: Montreal Neurological Institute
MRI: magnetic resonance imaging
OFC: orbitofrontal cortex
PET: positron emission tomography
VAS: visual analogue scale
